# Exploring Satisfaction and Migration Intentions of Physicians in Three University Hospitals in Poland

**DOI:** 10.3390/ijerph17010043

**Published:** 2019-12-19

**Authors:** Katarzyna Dubas-Jakóbczyk, Alicja Domagała, Dorota Kiedik, Juan Nicolás Peña-Sánchez

**Affiliations:** 1 Department of Health Economics and Social Security, Institute of Public Health, Faculty of Health Sciences, Jagiellonian University Medical College, 31-531 Krakow, Poland; 2 Department of Health Policy and Management, Institute of Public Health, Faculty of Health Sciences, Jagiellonian University Medical College, 31-531 Krakow, Poland; 3 Department of Economics and Quality in Health Care, Faculty of Health Sciences, Wroclaw Medical University, 50-367 Wrocław, Poland; dorota.kiedik@umed.wroc.pl; 4 Department of Community Health and Epidemiology, College of Medicine, University of Saskatchewan, Saskatoon, SK S7N5E5, Canada; juan.nicolas.ps@usask.ca

**Keywords:** university hospital, physician satisfaction, migration

## Abstract

Introduction: University hospitals constitute a unique group of health care organizations which traditionally link three functions: (1) providing highly specialized services, (2) teaching activities, and (3) conducting research. Objectives: To assess the level of carrier satisfaction among physicians working in three university hospitals in Poland (1); to assess whether the physicians have the intention to migrate and what the main reasons for migration are (2); and to identify the actions that might be taken at the hospital level to mitigate physicians’ intentions to migrate (3). Methods: Cross-sectional study with both quantitative and qualitative components. In the quantitative part, an online questionnaire was distributed among physicians working in three university hospitals. A total number of 396 questionnaires were analyzed. In the qualitative part, in-depth interviews with six hospital managers were conducted and analyzed using thematic analysis. Results: On a scale from one “very dissatisfied” to six “very satisfied”, the mean career satisfaction of physicians was 4.0 (SD = 0.74). The item with the lowest mean concerned salary level (2.8, SD = 1.41). In the sample, 34% of physicians declared intentions to migrate from Poland. The main reasons for the intention to migrate were: Better working conditions abroad, higher earnings, the ability to maintain better work-life balance, better training opportunities abroad, and problems due to a stressful current workplace. Hospital managers considered the actions that can be taken at the hospital level to mitigate physicians’ migration to be specific to those focused on the working environment. Conclusions: Career development opportunities and features related to the working environment are the main factors influencing physicians’ satisfaction and migration intentions that can be modified at the university hospital level.

## 1. Introduction

University hospitals constitute a unique group of health care organizations which traditionally link three functions: (1) providing highly specialized medical services, (2) teaching activities, and (3) conducting research [1,2,3]. University hospitals often represent the highest reference level of health care provision and deal with the most complex cases. They are involved in medical education activities at both the under and post-graduate levels. As a consequence, university hospitals are often the first entry point for medical students, where they gather their first experiences and their attitudes as prospective physicians are initially established [4]. Finally, these types of hospitals are actively engaged in research initiatives which provide unique scientific career development opportunities for their employees [5].

The university hospital setting is a particularly challenging and competitive work environment for medical doctors, involving patient care, training tasks, and clinical research. This work environment could be associated with unfavorable health effects [6,7], which highlights the need to assess and monitor physician satisfaction. Researchers in numerous countries have conducted studies focused on satisfaction [4,8,9] as well as stress and burnout among physicians working in university hospitals [10,11,12]. Physician satisfaction is a complex and multidimensional concept which can be affected by personal and external factors [13,14]. Physicians’ satisfaction has significant effects on their productivity, their work ability, and the quality of patient care, as well as their intentions to leave the job or their intent to continue practicing medicine [15,16,17].

In 2018 in Poland, there were 36 university hospitals owned and operated by 13 public medical universities. These hospitals are financed via mixed sources: By the public health insurance payer for medical service provision; by the Ministry of Health and Ministry of Science and Higher Education for didactic activities; and via diverse external sources for research activities. Physicians working in university hospitals are often employed on a double-position basis: By the hospital as a physician and by the university as a teacher and/or researcher. Most of the university hospitals in Poland are big and multidisciplinary medical centers. Among the 36 university hospitals functioning in 2018, 25 units (70%) had more than 500 hospital beds, while eight of them (22%) had more than 800 beds. University hospitals in Poland provide approximately 25% of all medical services and 75% of those are highly specialized [18]. These hospitals face challenges related to an unstable financial situation and, therefore, have strong pressure to conduct restructuration activities. Despite being discussed by policy-makers for many years, there are no specific regulations for university hospitals in Poland that, for example, control aspects of the high cost of didactic activities and treatments of the most complex patients [18,19]. Within the last couple of years, some medical universities have decided to merge and/or consolidate their hospitals to improve their financial situation and operational efficiency.

Polish university hospitals face another major challenge, the generally difficult condition of medical staff. The situation of Polish physicians is complex due to the identified shortages (Poland has the lowest ratio of physicians per one thousand population in the European Union) and age structure concerns: The mean age medical doctor was 50.2 in 2017 and 54.2 in the case of doctors with a specialization [20]. Working conditions are characterized by heavy workload, long working hours/dual practice, and a high burden of administrative tasks. Also, the migration of Polish physicians is a significant challenge. The exact scale of migration is unknown due to incomplete and insufficient data and the lack of mechanisms for monitoring this phenomenon [21]. The scale of migration is estimated based on the number of certificates issued by the regional chambers confirming professional qualifications that give the legal right to practice in other European Union countries. The total number of such certificates issued to Polish physicians between 2004 and 2017 was 9.535 (about 7% of physicians with the legal right to practice) [22]. The most popular destination countries are Germany, the United Kingdom, the Netherlands, and the Scandinavian countries [23].

There is an urgent need to better recognize the drivers of physician satisfaction and migration trends. If the determinants of migration are better known, proper actions can be developed to prevent physicians from leaving the Polish healthcare system. The recently published studies [24,25] provided important insight into the issue of satisfaction and migration intention among the general population of physicians working in hospitals in Poland. The aim of the current study was to focus solely on university hospitals and analyze the problem from the hospital managers’ perspective. The specific objectives were to: (1) assess the level of career satisfaction and related factors among physicians working in three university hospitals in Poland; (2) assess whether the physicians working in these hospitals have an intention to migrate and what the main reasons for migration are; and (3) identify the actions that might be taken at the hospital level to improve physicians’ career satisfaction and mitigate their intention to migrate.

## 2. Materials and Methods

A cross-sectional study with both a quantitative and a qualitative component was conducted. In the quantitative part, an online survey was distributed among physicians working in three university hospitals in Poland. In the qualitative part, in-depth interviews with hospital management representatives were conducted. The survey was conducted between March and June 2018 and the interviews were completed between January and February 2019. The ethical approval of the Jagiellonian University Bioethical Committee was obtained to conduct the quantitative part of the study (approval number: 122.6120.290.2016) while the qualitative component did not require the consent of this committee (in-depth interviews with administrative staff can be exempted).

### 2.1. Sample

University hospitals were selected based on the following criteria: (1) geographic area (hospitals located in three different Polish geographic areas situated in non-neighboring regions); (2) specific profile and size (multidisciplinary public university hospitals with more than 800 beds); and (3) reachable hospital managers to support and authorize conducting the study (written permission was obtained, signed by hospital directors of the included hospitals). Three public university hospitals were invited, and their directors agreed to participate.

In the quantitative part, all physicians working in the included hospitals were invited to participate by completing an on-line questionnaire. A total number of 408 physicians filled in the questionnaire (response rate 21%), yet in the data analysis we included only those with no missing data: 396 questionnaires. In the qualitative part, two representatives of the management of each hospital were both invited and subsequently agreed to participate in an in-depth interview. The participants’ inclusion criteria included: (1) holding a managerial position directly involved in human resources management and (2) a minimum of five years of working experience in the analyzed hospital. A total number of six interviews were conducted.

### 2.2. Quantitative Phase

Personalized e-mails were sent to physicians, providing the objective of the study, terms of anonymity and confidentiality, and a link to the online questionnaire. Three follow-up e-mails were sent. Moreover, paper copies of the survey were prepared and delivered to physicians who experienced problems completing the on-line version of the survey.

The online questionnaire had two parts. The first part was focused on the satisfaction of the given physician which was measured using the Polish version of a questionnaire designed and validated to measure physicians’ career satisfaction [26,27,28]. This 17-item career satisfaction questionnaire had been previously adapted and used in the Polish context [24,26]. The items of this instrument were measured on the Likert scale from one “very dissatisfied” to six “very satisfied”. Standardized levels of physicians’ career satisfaction were computed by summing the item scores and dividing them by 17 with standardized scores ranging from 1.0 to 6.0 [24,26]. The questionnaire to measure physicians’ satisfaction was shown to have good internal consistency reliability assessed by the Cronbach’s alpha coefficient (α = 0.906) [24]. The second part of the questionnaire was related to the intention to migrate which was assessed with the following question: “Are you currently considering practicing medicine abroad?” with four options: 1-“definitely no”, 2-“probably no”, 3-“probably yes”, or 4-“definitely yes”. Physicians who answered “probably yes” or “definitely yes” were classified as those with an intention to migrate. Only these respondents were asked about reasons for migration. This part of the questionnaire was developed based on a previous study completed in Ireland [17], adapted and validated for Polish settings [25]. The content validity of the questionnaire in Polish was previously evaluated by a committee of experts [26] and practicing physicians [25].

In statistical analyses, continuous variables were expressed as mean (standard deviation, SD) or as median (interquartile range, q1–q3), as appropriate. The Shapiro-Wilk test was used to assess conformity with a normal distribution. We tested the associations between the two primary outcomes —the standardized levels of satisfaction and the intention to migrate with physicians’ demographics and work-related variables. The continuous variables were compared between groups using a *t*-test or one-way analysis of variance (ANOVA, in the case of at least three groups) or the Mann-Whitney U test (Kruskal-Wallis test, in the case of at least three groups) for variables with non-normal distribution. Categorical variables were described by percentages and compared using the χ^2^ test. Unconditional and multivariable regression models were performed to investigate the relationships between the considered factors and intention to migrate. For the purposes of the regression analysis, a dichotomous variable was defined: Physicians who answered “probably yes” or “definitely yes” were classified as those with an intention to migrate and physicians who answered “probably no” or “definitely no” were grouped as those without an intention to migrate. A backward stepwise method was used to assed the significant predictors of intention to migrate. Odds ratios (ORs), with their corresponding 95% confidence intervals (CI), were computed and reported. Statistical analyses were performed using SPSS 23.0 (SPSS Inc., Chicago, IL, USA). *p*-values < 0.05 were accepted as statistically significant.

### 2.3. Qualitative Phase

The in-depth interviews were conducted after completion of the survey analysis. The outcomes of the questionnaire survey for the particular hospital were presented to the invited participants prior to the interview (the results were sent via email prior to the interview date). These reports presented the survey results for the particular hospital and the average for the whole sample. The choice of respondents was based on purposeful sampling—two managers from each included hospital were invited.

The hospital managers were interviewed based on a structured scenario. The scenario included: General guidelines for the interviewers (e.g., presenting the interview process and objectives; confirming its confidential character) and the set of questions to be asked. The questions were clustered around two main topics: (1) respondents were asked to comment on the outcomes of the survey for their hospitals (e.g., reasons for high/low level of physician satisfaction/intention to migrate) and (2) identify the actions that might be taken at the hospital level in order to improve physicians’ satisfaction and mitigate their migration intentions.

The interviews were conducted either by phone (two) or by direct face-to-face conversation (four); each respondent was interviewed individually. The interviews lasted a minimum of 45 min and were conducted in Polish by three researchers (the authors of this article). The interviews were recorded transcribed verbatim and verified by two researchers. The data were analyzed by two researchers using thematic analysis and following the stages as proposed by Braun & Clarke (2006) [29]. The inductive analysis approach and hand coding was applied. The processes of data gathering and analysis was iterative, the analysis started after conducting the first three interviews. The coding process, themes, and subthemes identification was done in Polish (the native language of both the respondents and the researchers involved). The final results were translated into English by one researcher and verified by the other one involved in data analysis.

## 3. Results

### 3.1. Quantitative Phase

Among the 396 participants, the mean age of the respondents was 42.3 years (SD = 11.9) and the majority of them were men (n = 207, 52%). In total, 259 physicians (65%) had a specialization and 285 (72%) performed additional shift-work duties. In terms of workload, 247 (62%) doctors were employed in other health care institutions and the mean number of working hours per week was 56.0 (SD = 17.9). The majority of the respondents (79%) were employed based on job agreement and 12% of doctors had additional managerial duties (e.g., ward chief) (Table 1).

The mean standardized level of physicians’ career satisfaction was 4.0 (SD = 0.74) on a scale from 1.0 to 6.0. The satisfaction items with the lowest scores were related to the salary levels (2.8, SD = 1.41), work-personal life balance (3.28, SD = 1.39), and the ability to maintain satisfying non-work related activities (3.34, SD = 1.38). Respondents reported the highest levels of satisfaction with the diversity of patients/their clinical conditions (4.6, SD = 0.96) and success in meeting the needs of their patients (4.5, SD = 0.98). Participants were also quite satisfied with the interactions/relationship with their direct supervisor (4.6, SD = 1.18), nurses (4.6, SD = 0.92), and other physicians (4.5, SD = 0.90) (Table 2).

Table 3 presents a comparison of the standardized levels of career satisfaction levels by demographic and job-related characteristics. There was a statistically significant difference in the career satisfaction levels between physicians with a completed specialization and those in a residency training, with specialized physicians reporting higher levels of career satisfaction (4.1 versus 3.8, *p* < 0.001). There was also a difference between physicians working exclusively in a university hospital and those also employed in other health care institutions (4.2 versus 3.9 respectively, *p* < 0.001). Also, physicians employed based on a contract formula reported a higher level of career satisfaction than those employed based on a job agreement or a mix of both forms (4.2 versus 3.9 and 3.8 respectively, *p* < 0.05).

Out of the 396 physicians who completed the questionnaire, 135 (34%) declared the intention to migrate: 24 (6%) who reported “definitely yes” and 111 (28%) “probably yes”. Among physicians with the intention to migrate (“definitely yes” and “probably yes”), the majority (n = 84, 62%) indicated a temporary character of the intended migration (they considered returning to Poland after few years). Germany, the United Kingdom, and the Scandinavian countries were the most often chosen destinations. The most frequently indicated reasons for migration (summary answers “agree” and “strongly agree”) were: Better working conditions abroad (99%); higher earnings abroad (98%); the ability to maintain better work-life balance abroad and better training opportunities abroad (93%); and problems due to the current workplace being stressful (89%) (Table 4).

Table 5 presents a comparison of the physicians with and without the intention to migrate and the demographic and job-related characteristics of the respondents. There was a statistically significant difference between physicians with and without the intention to migrate depending on the respondents’ age, gender, and whether or not they had children. Younger physicians, men, and those without children more often declared the intention to migrate. Also, physicians with the intention to migrate had a greater mean value of working hours per week than those without the intention to migrate. The latter included both hours working in the analyzed unit: 48.7 (SD = 12.6) versus 43.1 (SD = 15.1), *p* < 0.001 as well as total number of working hours: 61.7 (SD = 18.5) versus 53.1 (SD = 16.9), *p* < 0.001. Finally, the intention to migrate was associated with the overall level of career satisfaction, with physicians declaring the intention to migrate reporting lower level of career satisfaction: Mean 3.7 (SD = 0.78) versus 4.1 (SD = 0.68), *p* < 0.001.

Table S1 in the supplementary online material presents the results of unconditional and multivariable logistic regression. In the final multivariable model, the significant factors associated with the intention to migrate were age, gender, total number of working hours per week, and career satisfaction. Women were 59% less likely to migrate than men (OR = 0.41, 95% CI 0.24–0.68). The odds to migrate decreased by 0.08 (95% CI 0.90–0.94) and 0.55 (95% CI 0.31–0.63) per each additional year of age and unit of career satisfaction, respectively. In contrast, the number of working hours per week was positively associated with the intention to migrate. The odds of physicians’ intention to migrate increased by 0.02 (95% CI 1.01–1.04) per each working hour increase per week.

Respondents were also asked to indicate perceived barriers to migration. Most of the participants (67%) indicated ‘leaving family’ as a migration barrier. Almost 32% of respondents indicated a good professional position in Poland, while 20% and 14% indicated concerns related to the new working environment and foreign culture, respectively.

### 3.2. Qualitative Phase

Table 6 presents characteristics of the interview respondents. Three of them were female and three were men, and their years of working experience in the analyzed hospital ranged from five to 22 years. All hospital managers emphasized the importance of measuring and monitoring physicians’ satisfaction as a prerequisite for building effective human resources management strategies. In the opinion of most of the respondents, the factors that influence physician satisfaction and, in consequence, their migration intentions can be divided into two broad categories: Those which can be modified at the hospital level and those which should be applied at the system level (for the details of the thematic analysis results see the online supplementary material—Table S2). As a consequence, the actions that can be taken by the hospital managers themselves are rather limited to those directly related to the working environment.

At the hospital level, respondents emphasized the importance of direct and interpersonal relations between the employees. One of the managers pointed out: “*(…) in our hospital, we are trying to promote a culture of communication and building social networks among health care professionals*.” Few respondents indicated actions aimed at relieving physicians from administrative duties by increasing the number of support staff (e.g., medical assistants, medical secretaries, and care coordinators). Another factor mentioned by the respondents, which may contribute to improving physician satisfaction, and can be modified (at least partially) at the hospital level, is providing scientific career development opportunities (e.g., by providing administrative support for research grant application and realization). One of the respondents said: “*(…) many of our physicians are university professors, the top national researchers in their clinical fields; thus our obligation is to create the best environment in which they can conduct research and teach the younger generation of physicians*”.

While commenting on the outcomes of the questionnaire survey, three managers mentioned the challenges related to the generational gap between junior and senior physicians. One of the respondents said: “*(…) young physicians have a completely different set of values and attitudes than our senior staff, thus our management strategies must be modified accordingly, (…) at the same time we must ensure that our older doctors do not feel offended and/or disrespected by the ‘privileges’ given to the young*”.

In terms of the system level factors, the majority of the respondents emphasized that the issue of physicians’ salary levels is mainly outside their scope of influence. One of the respondents said: “*(…) in Poland the level of physicians’ salaries is not decided by the hospital manager, but government regulations, (…) and regardless of the recent increases—the money will never be enough to satisfy the growing expectations*”. Another respondent pointed out that a related issue is the practice of medical doctors working in multiple positions: “*(…) the fact that doctors are often dissatisfied with their work-life balance might not be related to their work in this particular hospital, but the fact that they often have additional jobs (in other hospitals or out-patient units)*”. Respondents highlighted the need of a system level strategy for medical staff remuneration issues as well as dedicated regulations for university hospitals, which would consider their special role within the system.

## 4. Discussion

Our results show that physicians working in the three analyzed university hospitals are rather satisfied with their careers. In the sample, the migration intention was 34%. Level of salary was most often indicated by the physicians as the main reason for dissatisfaction, as well as the reason for migration intention. There are, however, numerous factors that contribute to both the level of satisfaction and the migration intentions. These factors involve for example: Career development opportunities and different features related to the working environment, including relationships with co-workers. Junior physicians more often reported low levels of career satisfaction, as well as intention to migrate in comparison to those with a specialization. In the opinion of the hospital managers, the actions that can be taken at the hospital level aimed at improving physicians’ satisfaction are limited to those related to the working environment.

The results of our research are consistent with other studies measuring satisfaction of physicians working in European hospitals. According to the findings of a systematic review conducted in this field, physician satisfaction of those working in European hospitals is moderate and the majority of them reported being somewhat satisfied or very satisfied [30]. Doctors who participated in our study reported a high level of satisfaction with their interactions/relationships with direct supervisors, nurses, and other physicians. There is considerable evidence regarding the relationship between colleague support/team climate and physician satisfaction. Findings from numerous European studies reported that positive team climate and support from colleagues is associated with significantly higher job satisfaction [4,31,32,33].

In terms of the salary level being the reason for the biggest dissatisfaction, the context of the Polish health system needs to be taken into consideration. Economic factors are the most often indicated reasons for migration of medical staff [34] and higher earnings abroad was also indicated by our respondents as the main migration driver. In Poland, within the last two decades, the issue of medical workers’ salaries level has been the subject of numerous government regulations [35]. In 2017, after a series of junior physician strikes, the government adopted a regulation which would gradually increase medical workers’ minimum wage [36]. Despite the fact that average salaries of medical workers in Poland have increased during the last decade, physicians’ wages in Poland are still low in comparison with other high-income countries. In 2016, the average specialist salary was at the level of 1.4 times the average wage in the country, which was the lowest among the 21 OECD countries for which data are available [37].

Our respondents reported low satisfaction with work-personal life balance. This problem has also been shown to have a significant impact on physician satisfaction in other European studies [38,39]. In Poland, this issue might be related to physicians working simultaneously in multiple positions—62% of the questionnaire survey respondents reported also working in other health care institutions. In order to improve that situation in 2018, the Ministry of Health introduced an amendment to the regulations on physicians’ wages which provides financial incentives to work solely in one hospital [40]. This regulation covers only medical doctors employed based on a job agreement and offers a salary increase once the ‘loyalty agreement’, is signed.

The frequency of migration intention among physicians working in university hospitals was slightly higher than in general population of Polish physicians [25]. This can be explained by the lower mean age of the sample. Hospital managers pointed out that the reason for junior physicians being less satisfied and more willing to migrate than senior physicians might be related to the generational gap between these two groups. This constitutes a universal, global challenge and needs special attention and further in-depth analyses aimed at building effective health workforce management strategies. Numerous researchers conclude that the current generation of young medical doctors value their personal life and work balance as a high priority; they want better flexibility of working conditions and shorter hours [41,42,43]. This has profound consequences for the hospital management in terms of both recruiting and retaining young doctors, as well as running a multigenerational workforce environment [44,45]. One of the consequences of the above is that focusing solely on financial compensation aspects might be a misleading strategy to keep junior physicians from leaving. This is especially important in the context of the Polish health system, where after the 2017 series of junior doctor strikes, the government introduced so called ‘patriotic vouchers’, which are a guaranteed salary increase for physicians who commit to staying in Poland for a minimum of two years after completing residency training [40].

Finally, our results suggest that physician satisfaction is negatively related to the intention to migrate: A higher level of satisfaction is associated with a lower willingness to migrate. Therefore, special attention of healthcare managers should be devoted to measurement and improvement of physicians’ satisfaction. Numerous European studies reported that physicians’ dissatisfaction with working conditions could be an emigration driver [46,47,48]. In terms of the migration problem, the vast majority of the study participants with intention to migrate indicated, inter alia, better training opportunities abroad as a reason for migration (57% respondents “strongly agreed” and 36% “agreed”). This provides an important insight for university hospital managers, as these types of hospitals are, by their statutory functions, responsible for medical doctor training and have much broader capacities in this field than other in-patient care institutions.

The main limitation of the study’s quantitative component is the relatively low response rate for the survey questionnaire, which may limit the generalizability of the results. However, numerous international studies have indicated that surveys among physicians have low response rates if compared with the general population [49,50,51] and the key challenge with surveying physicians is low response rate [52,53]. In Poland, according to the information provided by the National Chamber of Physicians (NCP) the willingness of physicians to participate in surveys is very low [54]. In a 2018 survey conducted by the NCP only 2.7% doctors to whom the invitations had been sent, visited the webpage with the questionnaire while only 12% of those who visited, actually opened the questionnaire [54]. We expect that one of the reasons for limited participation in our survey was the high workload of physicians as well as also including in the targeted population physicians employed on a part time basis, who therefore have relatively low participation in hospital related activities. In addition, there is evidence that medical doctors do not significantly differ among respondents and non-respondents in terms of answers and group characteristics and larger sample sizes compensate for greater nonresponse [53]. Yet, the important challenge for the researchers is increasing the number of physicians participating in the future study.

In the case of the qualitative component, the main limitations are related to purposeful sampling and the relatively low number (six) of the in-depth interviews. These may impact the representative nature of the sample as well as contribute to researcher bias. Yet, in the case of our study—the interviews were aimed, inter alia, at a deeper understanding of the questionnaire survey results and were thus limited to the managers of the three university hospitals. All participants had similar professional backgrounds and research suggests that in the case of the participant homogeneity a smaller sample can be chosen in order to determine the main aspects of the analyzed problem [55]. We believe we reached data saturation after completing the sixth interview (the codes were repetitive and the main themes were clearly identified). This is in line with consensus theory according to which respondents who are experts in a particular field tend to agree more with each other and data saturation can be achieved with a smaller number of participants [56]. The researcher bias was minimalized by following a strict methodological approach.

The main implication of our study is the importance of measuring and monitoring physician satisfaction. The results have provided an important insight for hospital managers, as well as for health policy makers on the factors that contribute to physicians’ satisfaction and migration intentions. Our work can help in developing effective hospital strategies aimed at improving physicians’ working and employment conditions (e.g., better team working and more flexible arrangements), and, in the context of the migration problem, to retain medical doctors in the country. At the system level, two issues seem crucial. First is the need for transparent and fair organization of physician salary regulations, which in recent years has been an effect of rather ad hoc decisions rather than any form of national strategy [35]. The second issue is related to improving physicians’ training opportunities, which was one of the postulates of the above mentioned junior medical doctors strikes. In the case of the latter, in 2018 the representatives of the National Chamber of Physicians announced that they were launching work on proposing changes to the dedicated regulations [57].

## 5. Conclusions

Physicians working in university hospitals in Poland are rather satisfied with their careers. Level of salary was most often indicated as the reason for dissatisfaction and as a reason for migration intention. There are, however, other factors that contribute to both the level of career satisfaction and migration intentions; for example, working environment and career development opportunities. Junior physicians more often reported lower levels of satisfaction, as well as a high intention to migrate, in comparison to physicians with a specialization. Hospital managers should pay special attention to improving working conditions and providing better career and training development opportunities for physicians.

## Figures and Tables

**Table 1:** Descriptive statistics.

Variable	n = 396
Age, years, mean (SD)	42.34 (11.96)
Men, n (%)	207 (52%)
Marital status (in a relationship), n (%)	306 (77%)
Have children, n (%)	264 (67%)
Specialist, n (%)	259 (65%)
Work experience, years, median (q1–q3)	15 (6–26)
Additional shift-work duties, n (%)	285 (72%)
Managerial duties in hospital, n (%)	46 (12%)
Number of working hours in hospital per week, mean (SD)	45.0 (14.5)
Employment in other health care institution, n (%), including:	247 (62%)
Additional out-patient care provider, n (%)	189 (48%)
Additional in-patient care provider, n (%)	83 (21%)
Total number of working hours per week, mean (SD)	56.0 (17.86)
Type of employment ^†^, n (%)	
Job agreement	310 (78.5%)
Contract	72 (18.2%)
Mix	13 (3.3%)

^†^ The three forms include: Job agreement (usually permanent, based on salary); contract (temporary, based on fee for service); mix (physician is employed both based on a job agreement and by contract); and data are shown as mean (standard deviation = SD), median (q1–q3), or number (percentage).

**Table 2:** Mean levels of satisfaction, with corresponding standard deviations (SD) and medians per each of the items of the career satisfaction questionnaire (n = 396).

No.	Item of Career Satisfaction	Mean	SD	Median
1	your interactions and relationship with other physicians	4.51	(0.90)	5
2	the doctor-patient relationships	4.41	(0.84)	4
3	the diversity of patients you see (and their clinical conditions)	4.61	(0.96)	5
4	your success in meeting the needs of your patients	4.53	(0.98)	5
5	your ability to access resources needed to treat your patients	3.81	(1.20)	4
6	your capacity to keep up with advances in your clinical specialty	4.00	(1.19)	4
7	your role in organizing prophylactic programs for patients	3.49	(1.09)	4
8	your interactions and relationship with nurses	4.55	(0.92)	5
9	your interactions and relationship with the hospital administration	3.57	(1.26)	4
10	your interactions and relationship with your direct supervisor	4.60	(1.18)	5
11	your authority to get your clinical decisions carried out	4.40	(1.11)	5
12	your ability to control your work schedule	3.97	(1.23)	4
13	your work-personal life balance	3.28	(1.39)	3
14	your salary	2.80	(1.41)	3
15	your career advancement	4.00	(1.15)	4
16	planning of your career advancements	3.88	(1.15)	4
17	your ability to maintain satisfying non-work-related activities (e.g., social and cultural activities)	3.34	(1.38)	3

**Table 3:** Comparison of the levels of career satisfaction between physicians with different demographic and job-related characteristics.

Variable		Satisfaction Level		
	n	Mean	SD	*p* Value
Gender, n (%)				
men	207	4.05	(0.75)	
women	189	3.91	(0.72)	0.065 ^A^
Marital status, n (%)				
single	90	3.98	(0.73)	
in relationship	306	3.99	(0.74)	0.98 ^A^
Children, n (%)				
no	132	3.95	(0.77)	
yes	264	4.00	(0.72)	0.54 ^A^
Specialist, n (%)				
no	137	3.78	(0.78)	
yes	259	4.09	(0.69)	<0.001 ^A^
Additional shift-work duties, n (%)				
no	111	4.09	(0.72)	
yes	285	3.94	(0.74)	0.07 ^A^
Employment in other healthcare institution, n (%)				
no	149	4.17	(0.69)	
yes	247	3.87	(0.74)	<0.001 ^A^
Type of employment, n (%)				
job agreement	310	3.94	(0.77)	
contract	72	4.22	(0.54)	
mix	13	3.77	(0.74)	0.0085 ^B^

^A^*p*-value from *t*- test, ^B^*p*-value from analysis of variance (ANOVA).

**Table 4:** Distribution of answers on reasons to migrate among physicians with the intention to migrate (% of answers).

Reason/% of Answers	Strongly Disagree	Disagree	Agree	Strongly Agree
Hours too long	7.4	20.7	34.8	37.0
Non-core tasks	3.7	13.3	23.7	59.3
Understaffed	2.2	11.9	34.1	51.9
Employer doesn’t support	7.4	26.7	37.8	28.1
Supervisor doesn’t support	27.4	40.7	21.5	10.4
Not respected	32.6	45.2	16.3	5.9
Quality of training poor	6.7	29.6	43.0	20.7
Choice of training limited	8.9	32.6	37.0	21.5
Training not satisfactory	8.9	28.9	37.8	24.4
Career progression limited	3.7	22.2	43.0	31.1
Work environment stressful	3.0	8.1	37.0	51.9
Earn more	0.0	2.2	18.5	79.3
Better training opportunities abroad	0.7	5.9	36.3	57.0
Migrate to be competitive	9.6	46.7	28.9	14.8
Better working conditions abroad	0.0	0.7	23.7	75.6
Better work-life balance	1.5	5.2	28.9	64.4
Family/personal reasons	41.5	40.0	14.8	3.7

**Table 5:** Comparison of the physicians with and without the intention to migrate and different demographic and job-related characteristics.

Variable	Migration Intention				*p* Value
	No		Yes		
Age, mean (SD)	45.4	(12.2)	36.5	(9.0)	<0.001 ^A^
Gender, n (%)					
men	125	(60.4%)	82	(39.6%)	
women	136	(72.0%)	53	(28.0%)	0.015 ^B^
Marital status, n (%)					
single	52	(57.8%)	38	(42.2%)	
in relationship	209	(68.3%)	97	(31.7%)	0.06 ^B^
Children, n (%)					
no	71	(53.8%)	61	(46.2%)	
yes	190	(72.0%)	74	(28.0%)	<0.001 ^B^
Specialist, n (%)					
no	62	(45.3%)	75	(54.7%)	
yes	199	(76.8%)	60	(23.2%)	<0.001 ^B^
Work experience, years, median (q1–q3)	19	(8–30)	7	(4–18)	<0.001 ^C^
Type of employment, n (%)					
job agreement	202	(65.2%)	108	(34.8%)	
contract	51	(70.8%)	21	(29.2%)	
mix	8	(61.5%)	5	(38.5%)	0.62 ^B^
Additional shift-work duties, n (%)					
no	84	(75.68%)	27	(24.32%)	
yes	177	(62.11%)	108	(37.89%)	0.01 ^B^
Number of working hours in hospital per week, mean (SD)	43,1	(15.1)	48.7	(12.6)	<0.001 ^A^
Employment in other healthcare institution, n (%)					
no	105	(70.47%)	44	(29.53%)	
yes	156	(63.16%)	91	(36.84%)	0.14 ^B^
Total number of working hours per week, mean (SD)	53.1	(16.9)	61.7	(18.5)	<0.001 ^A^
Career satisfaction, mean (SD)	4.12	(0.68)	3.7	(0.78)	<0.001 ^A^

^A^*p*-value from *t*-test, ^B^*p*-value from χ^2^ test, and ^C^ *p*-value form Mann-Whitney U test.

**Table 6:** Characteristics of the in-depth interviews of the respondents.

No	Hospital	Position	Age (Years)	Gender	Employment in Hospital (Years)
1.	Hospital A	Director for medical affairs	49	F	20
2.	Hospital A	Chief of the ward	52	M	22
3.	Hospital B	Director for quality	55	F	12
4.	Hospital B	Chief of the ward	60	M	25
5.	Hospital C	Coordinators of hospital’s clinics	58	F	21
6.	Hospital C	Chief of the ward	48	M	5

## Supplementary Materials

The following are available online at https://www.mdpi.com/1660-4601/17/1/43/s1, Table S1: Unconditional and multivariable logistic regression models predicting migration intention. Table S2: Themes identified in thematic analysis of the interviews.
