# Supplementary material for: Exploring Satisfaction and Migration Intentions of Physicians in Three University Hospitals in Poland
Source: Int J Environ Res Public Health. 2019 Dec 19;17(1):43. doi: 10.3390/ijerph17010043 (PMC6981599; doi:10.3390/ijerph17010043)
Supplement: Supplementary file 1 [file ijerph-17-00043-s001.pdf]

SUPPLEMENTARY MATERIAL

Table S1. Unconditional and multivariable logistic regression models predicting migration intention

Variable		Unadjusted OR[A] (95% CI)	Adjusted OR[B] (95% CI)
Age		0.93 (0.91-0.95)	0.92 (0.90-0.94)
Gender,			
	male	1	1
	female	0.59 (0.39-0.91)	0.41 (0.24-0.68)
Marital status,			
	single	1	
	in relationship	0.64 (0.39-1.03)	
Having children,			
	no	1	
	yes	0.45 (0.29-0.70)	
Work experience, years,		0.93 (0.91-0.95)	
Specialist,			
	no	1	
	yes	0.25 (0.16-0.39)	
Type of employment,			
	job agreement	1	
	contract	0.77 (0.44-1.35)	
	mix	1.17 (0.37-3.66)	
Additional shift-work duties,			
	no	1	
	yes	1.90 (1.15-3.12)	
Total no of working hours in hospital per week,		1.03 (1.01-1.04)	
Number of employment places,			
	only 1 hospital	1	
	also additional providers	0.97 (0.32-2.88)	
Total no of working hours per week,		1.03 (1.02-1.04)	1.02 (1.01-1.04)
Career satisfaction,		0.46 (0.34-0.63)	0.45 (0.31-0.63)

OR – odds ratio, A – unconditional logistic regression models, B - final multivariable logistic regression model

Table S2. Themes identified in thematic analysis of the interviews

No	Theme	Subthemes
1.	The importance and challenges of measuring physician satisfaction	• measuring and monitoring physicians' needs and opinions;<br>• standard questionnaire vs. special character of university hospitals;<br>• physicians' aversion to any form of additional administrative tasks;
2.	Hospital level factors influencing physician satisfaction and migration intentions	• importance of interpersonal relations between employees;<br>• burden of administrative duties;<br>• carrier development opportunities - special character of university clinics;<br>• introduction of support staff/allied personnel
3.	Generational gap between physicians	• different set of values/attitudes held by junior and senior doctors;<br>• the need to apply different management strategies;
4.	System level factors influencing physician satisfaction and migration intentions	• most important factors being beyond the hospital mangers' influence;<br>• regulatory wage increases – ad hoc government actions;<br>• level of salaries – still too low;<br>• culture of working in multiple positions;
5.	Special character of working in university hospitals	• lack of dedicated regulations for university hospitals;<br>• prestige of working in university clinic vs. workload related to the different roles and tasks of medical staff;<br>• leaving the university hospitals and seeking employment in outpatient clinics –the scale and reasons for the problem.
